# Sleep of recruits throughout basic military training and its relationships with stress, recovery, and fatigue

**DOI:** 10.1007/s00420-022-01845-9

**Published:** 2022-02-28

**Authors:** Sean Bulmer, Brad Aisbett, Jace R. Drain, Spencer Roberts, Paul B. Gastin, Jamie Tait, Luana C. Main

**Affiliations:** 1 School of Exercise and Nutrition Sciences, Deakin University, Centre for Sport Research, Geelong, VIC Australia; 2 School of Exercise and Nutrition Sciences, Deakin University, Institute for Physical Activity and Nutrition, 221 Burwood Highway, Burwood, Geelong, VIC 3125 Australia; 3 Defence Science and Technology Group, Fisherman’s Bend, Melbourne, Australia; 4 School of Allied Health, Human Services and Sport, La Trobe University, La Trobe Sport and Exercise Medicine Research Centre, Melbourne, VIC Australia

**Keywords:** Army, Stress, Recovery, Recruits, SRSS, Sleep, Well-being, Workload

## Abstract

**Objective:**

Studies in basic military training (BMT) examining sleep are largely cross-sectional, and do not investigate relationships between sleep, stress, recovery and fatigue. The aims of this study were to (1a) quantify changes in recruits’ sleep quantity and quality over 12 weeks of BMT; (1b) quantify changes in recruits’ perceptions of stress, fatigue and recovery over BMT; and (2) explore relationships between sleep, and perceptions of stress, fatigue and recovery.

**Methods:**

45 recruits (37 male; 8 female, age: 25.2 ± 7.2 years, height: 176.2 ± 10.0 cm, mass: 76.8 ± 15.0 kg) wore ActiGraph GT9X’s for 12 weeks of BMT, collecting sleep duration, efficiency and awakenings. Subjective sleep quality, fatigue were measured daily, with stress and recovery measured weekly. Multi-level models assessed relationships between sleep, and stress, recovery, and fatigue.

**Results:**

Objective daily means for sleep duration were 6.3 h (± 1.2 h) and 85.6% (± 5.5%) for sleep efficiency. Main effects were detected for all mean weekly values (*p < *0.05). Sleep quality showed the strongest relationships with stress, recovery and fatigue. The best model to explain relationships between, stress, recovery and fatigue, included sleep quality, sleep duration, sleep efficiency and awakenings.

**Conclusions:**

The reported mean sleep duration of 6.3 h per night may negatively impact training outcomes across BMT. Combining both subjective and objective measures of sleep best explained relationships between sleep metrics stress, fatigue and recovery. Perceived sleep quality was most strongly related to change in stress, recovery, or post-sleep fatigue.

## Introduction

Sleep plays a fundamental role in physical adaptation (Vitale et al. 2019), cognition and the consolidation of new skills (Scott et al. 2006), mental health and wellbeing (Lentino et al. 2013); as well as injury and illness prevention (Halson 2014; Szivak and Kraemer 2015). Noting that changes to sleeping environment, sleep schedule, and periods of deliberate sleep restriction are inherent to basic military training (BMT) (Adrian et al. 2019; Crowley et al. 2012), insufficient sleep during BMT may compromise recruit training outcomes and health (Belenky et al. 2003; Dattilo et al. 2011). During focus group discussions U.S. recruits reported their sleep during BMT fell to < 6 h, with poorer quality, compared to ≥ 8 h of sleep at home (Crowley et al. 2012). Similarly, pre-and-post BMT questionnaires indicated that 13% of British recruits slept < 7 h pre BMT, increasing to 38% when assessed post BMT (Wentz et al. 2018). However, these studies have examined sleep using cross-sectional study designs and have not captured serial sleep measurements throughout training (Crowley et al. 2012; Wentz et al. 2018). Without a more complete understanding, training staff cannot accurately manage sleep or provide interventions across BMT to improve health and training outcomes if required.

Symptoms of fatigue are inherent to BMT due to the often stressful, and highly physiologically challenging training environment (Shattuck et al. 2018). A combination of high physical and mental fatigue can increase the risk of musculoskeletal injury, which are common in BMT (Knapik 2014). A 2018 review of 29 studies concluded that mental fatigue degrades endurance, motor skill and decision-making performance in athletes (Pageaux and Lepers 2018), all of which are common requirements throughout BMT. Additionally, soldiers in fatigued states suffer from reduced visual vigilance, decision-related reaction time, short-term memory, navigation skill and in some cases marksmanship accuracy through training exercises (Lieberman et al. 2016a; Lieberman et al. 2006). Given the associated reduction in training and performance with fatigue, identifying where and when recruits experience excessive fatigue could help identify key recovery points. However, existing studies typically provide only isolated fatigue measurements pre-, during or post-BMT (Lieberman et al. 2016b). Serial measurement of fatigue across the course of BMT would overcome these limitations and inform empirical evidence-based fatigue monitoring and recovery prescription.

Higher perceptions of stress early in BMT (i.e. first quarter) predict all-cause attrition in British junior entry recruits with 85% accuracy (Jackson et al. 2011). However, regular measurements of perceived stress (as distinct from physical or physiological training stress) across BMT and similar military settings have not been investigated. When an individual perceives high stress, and where current pressures and demands feel unmanageable, decision-making and behavioural processes are altered, due to increased circulation of adrenaline and cortisol, which increase arousal and alertness (Russell and Lightman 2019). This heightened state is desirable during danger and to alleviate fatigue for acute bouts of performance, although elevated arousal can be detrimental to normal physiological and cognitive functioning if sustained or during periods where such high stimulation is not required (Russell and Lightman 2019). In recruit and cadet populations, this phenomenon has been demonstrated as higher levels of perceived stress are linked with depressed mood states, poor academic and physical task performance, and negative sleep outcomes during key periods of training (Crowley et al. 2012; Miller and Shattuck 2005). An assessment of stress over entire training periods is important given that stress can accumulate over time, and chronic stress is known to compromise effective execution of duty (Tucker et al. 2009). However to date, no study has attempted to evaluate changes in perceived stress across a BMT program via serial measurement.

Recovery strategies (e.g., physical rest, increased sleep) can help to counter some of the effects of sleep loss, stress and fatigue induced by BMT (Caravalho 2015), however, optimal recovery requirements are often highly individual (Kellmann et al. 2018). Poor recovery practice can contribute to overtraining, reduced performance capacity, and injury (Kellmann et al. 2018), all of which are noted outcomes during BMT (Booth et al. 2006; Piirainen et al. 2019). Whether an individual feels that sufficient recovery has occurred could be valuable when contextualising the current stress and fatigue state. In exercise prescription, recovery is a fundamental principle of training, with periods of recovery between exposure to both acute and chronic training stimuli required for positive adaptation (Nässi et al. 2017). The importance of recovery and the periodisation of exercise programs using dedicated recovery periods has been established in athletic contexts (Kellmann et al. 2018). However, investigation into recovery as distinct to fatigue is uncommon in military settings. Periodised physical training in the military has occurred increasingly over the past two decades (Schiotz et al. 1998), but there is only recent evidence of its application to an entire military training program (Abt et al. 2016). Therefore, although adequate recovery is required for physical adaptations to occur, perceptions of recovery during BMT are largely untested.

Findings from other physically demanding occupations have observed relationships between sleep, fatigue and stress (Kellmann et al. 2018; Nässi et al. 2017; Wolkow et al. 2015b). However, these relationships are rarely clear-cut, and are influenced by many factors including the training setting and individual mental and physical characteristics. While monitoring of BMT recruits is increasing in empirical research, assessment of relationships between sleep and stress, fatigue, and recovery during BMT are yet to be systematically quantified across the entirety of BMT. This would be a first step toward a sleep, fatigue and recovery management strategy for recruits, as the cohort must first be observed, before deeper investigation is undertaken. With this in mind, the aims of this study were to (1a) quantify changes in recruits’ sleep quantity and sleep quality over 12 weeks of BMT; (1b) quantify changes in recruits’ perceptions of stress, fatigue and recovery over the 12 weeks of BMT; and (2) explore relationships between sleep, and perceptions of stress, fatigue and recovery. It was hypothesised that recruits would experience high levels of stress and fatigue, and impaired sleep and recovery, during BMT.

## Methods

### Recruitment and participants

This study was part of a larger project designed to quantify training loads and monitor the physiological and psychological responses of Australian Army recruits undertaking the 12-week BMT program at the Army Recruit Training Centre, Blamey Barracks, Kapooka, Australia. To contextualise results, Table 1 displays the focus of each week of training across the program. Power analyses were undertaken for the entire suit of measures included in the wider study. Inflammatory biomarkers, as presented in Tait et al. (2021), have the smallest measurable change of all outcome measures included in the wider study, and so this outcome measure was used to determine the required sample of *N* = 24. To account for up to 50% all cause attrition, an initial recruitment of *N* = 48 was sought. The risks associated with participation were verbally explained to recruits, and a written project description was provided by an investigator at a group briefing as approved by the Departments of Defence and Veterans’ Affairs Human Research Ethics Committee (protocol number: 021-17). Uniformed military staff were not present for the briefing, and it was made clear that participation was voluntary. Anthropometric data from Army’s initial screening of the recruits were provided. Forty-five recruits gave written, informed consent to participate (37 male; 8 female, age: 25.2 ± 7.2 years, height: 176.2 ± 10.0 cm, mass: 76.8 ± 15.0 kg). This ratio of male to female recruits is representative of current intakes while the Australian Army aims to increase female recruitment. All participants met the enlistment minimum fitness requirements of 45 sit-ups with feet held, level 7.5 on the Beep Test (i.e., a multi-stage shuttle run test) and 8 (female) or 15 (male) push-ups.

**Table 1:** Changes in recruit workload according to training program summary across the 12-week BMT program

Week	1	2	3	4	5	6	7	8	9	10	11	12
Class focus	Introduction	Team building	Military skills	Shooting introduction	Shooting consolidation	Theory review	Navigation	Medical	Battle theory	Field training	Field training	Revision
Physical training	Easy	Easy	Moderate	Firm	Hard	Recovery	Moderate	Firm	Firm	Hard	Hard	Recovery

### Objective sleep measurement

An ActiGraph GT9X (ActiGraph, Pensacola, FL, US) was worn on the non-dominant wrist 24 h per day for the entire 12 weeks, apart from Monday nights when devices were collected for data download and charging and returned to recruits Tuesday morning. Upon completion of the study, raw data files were analysed using the manufacturer’s propriety software (Actilife v6.13; ActiGraph, Pensacola, FL, US) and the Cole–Kripke sleep/wake detection algorithm (Cole et al. 1992). This algorithm has demonstrated 88% agreement (i.e., percentage of sleep and wake epochs correctly identified) when compared with polysomnography. Actigraphy has been previously used to assess the sleep of military personnel (Harris et al. 2015; Suurd Ralph et al. 2017). In accordance with guidelines (Ancoli-Israel et al. 2015), bed- and get-up times were recorded in a sleep diary to crosscheck sleep and wake states identified via the Cole-Kripke algorithm (Cole et al. 1992). The following sleep-related variables were recorded for all sleep periods: sleep duration (h; total duration of sleep obtained during the sleep period); sleep efficiency (%; total sleep time expressed as a percentage of time in bed); and awakenings (the number of movements per sleep period that are large enough to constitute a ‘potential’ awakening from sleep. This measure does not indicate increased cognitive activity or awaking to consciousness). Note that timing of participant consent and ActiGraph briefing meant that week-1 actigraphy was of limited duration and compliance. These data were not an accurate representation of the entire week and was not included in analyses.

Participants typically had one overnight sleep opportunity per 24 h throughout the monitoring period and from these, weekly means were calculated and used in analysis. However, during weeks 10 and 11, participants occasionally undertook night duties as part of field exercise training, which meant that overnight sleep was often bi-phasic. To accurately evaluate sleep on these nights, sleep duration, wake after sleep onset and awakenings were aggregated per 24 h, while sleep efficiency and sleep onset latency were averaged across both recorded sleep opportunities.

### Subjective sleep measurement

Subjective sleep quality was reported via pen and paper, on a 5-point Likert scale (1 “Very good” to 5 “very poor”). Note that higher scores equate to poorer sleep quality. While lengthier validated scales for measuring sleep quality over extended periods exist (Buysse et al. 1989), for the daily assessment of sleep in the current study, it was important to maintain as brief contact periods as possible to minimise participant burden. Therefore, this scale was chosen for brevity, with responses provided for the previous 24 h sleep each evening similar to work with wildfire firefighters (Vincent et al. 2016). Responses to all questionnaires in the study were transposed from pen and paper into Microsoft Excel. Data was double entered by two staff. Where discrepancies occurred, data was double checked for confirmation of correct value.

### Perceived stress, recovery, and fatigue

The Short Recovery Stress Scale (SRSS) assesses both recovery and stress states of an individual at the time of surveying, through eight questions (Nässi et al. 2017). The stress-related questions assess muscular stress, lack of activation, negative emotional state, and overall stress. Each item is also supported with examples that further clarify the description. The recovery-related questions assess physical performance capability, mental performance capability, emotional balance, and overall recovery. Participants were instructed to indicate the extent to which each statement applies to them at the time of surveying. Response options range from 0 (does not apply at all) to 6 (fully applies) (Nässi et al. 2017). Response scores for each question are summed to create a separate overall recovery and stress total score. This questionnaire has been found valid and reliable (Cronbach’s Alpha ≥ 0.74) in testing of stress and recovery. As a measure of daily fatigue throughout the week, a separate arbitrary pre- and post-sleep fatigue response was recorded daily on a 7-point Likert scale based on the Samn Perelli fatigue scale (“Fully alert, wide awake” to completely exhausted) (Samn and Perelli 1982). Weekly means were calculated from daily responses and used in analysis.

#### Data processing and statistical analysis

Statistical analyses were completed in SPSS software (IBM SPSS Statistics for Windows, Version 26.0, 2019, Armonk, New York). For aims 1a and 1b, each sleep variable, as well as SRSS component was assessed through within-participant analysis of variance (ANOVA), with repeated measures for week. When Mauchly’s test of sphericity indicated a violation (*p < *0.001), multivariate tests are described with Huynh–Feldt correction to account for sphericity and variation between participants across the analysis, as Huynh–Feldt is a conservative significance estimating approach (Fields 2013). Post hoc analyses were conducted using Bonferroni adjustment.

Multi-level Linear Models (MLM) assessed potential relationships between sleep, and subjective stress, recovery and fatigue. MLMs account for potential autocorrelation in the repeated measurements of each participant, as well as serial correlation of data points over time (Molenberghs and Verbeke 2001). Where a main effect for time was found, ‘timepoint’ was included as an independent variable within the MLM. These are appropriate for longitudinal repeated measures in which several factors are effects found ‘within-participants’. All sleep variables were entered individually into the models to identify any relationships with the single sleep variable and measures of stress, recovery and fatigue across the 12 weeks. Models were then iteratively built to achieve the lowest Akaike’s Information Criterion (AIC) possible, indicating highest goodness of fit. For each dependant variable (stress, recovery, pre-sleep fatigue and post-sleep fatigue), the model with the lowest AIC was deemed to best mediate sleep’s effect on that dependant variable. The three most effective models are presented as β-coefficients with standard deviation (SD), 95% confidence interval (CI), statistical significance within the model, and intercept for effects for each model (Table 2) (Wolkow et al. 2015a). Two models were equally effective for Pre-sleep fatigue, so both are reported. Some sleep variables included in a model structure did not have an individual statistically significant effect (*p* > 0.05). However, where the inclusion of a non-significant variable improved the AIC, that variable was deemed to provide a useful mediation of other independent variables, and therefore, was retained in the model.

**Table 2:** Parameters of multi-level linear models relationships for stress, recovery and fatigue relationships with sleep parameters

	Model	Parameter	AIC	β estimate (std. error)	95% CI Lower	95% CI Upper	*p*
SRSS stress	1	Intercept	2353	5.672 (0.946)	3.809	7.535	** < 0.001**
		Timepoint		− 0.088 (0.040)	− 0.167	− 0.008	**0.030**
		Sleep Quality		0.606 (0.267)	0.080	1.131	**0.024**
	2^a^	Intercept	1820	21.996 (6.638)	8.941	35.052	**0.001**
		Timepoint		− 0.084 (0.051)	− 0.184	0.015	0.097
		Sleep Quality		0.865 (0.317)	0.242	1.489	**0.007**
		Efficiency		− 0.213 (0.081)	− 0.373	− 0.053	**0.009**
		Duration		0.011 (0.008)	− 0.004	0.026	0.149
		Awakenings		− 0.173 (0.052)	− 0.274	− 0.072	**0.001**
	3	Intercept	1836	26.878 (6.413)	14.264	39.492	** < 0.001**
		Timepoint		− 0.083 (0.051)	− 0.183	0.017	0.103
		Efficiency		− 0.248 (0.081)	− 0.407	− 0.090	**0.002**
		Duration		0.013 (0.008)	− 0.002	0.028	0.083
		Awakenings		− 0.188 (0.051)	− 0.288	− 0.088	** < 0.001**
SRSS recovery	1	Intercept	2416	15.857 (0.990)	13.906	17.808	** < 0.001**
		Timepoint		0.018 (0.043)	− 0.067	0.104	0.673
		Sleep Quality		− 1.024 (0.285)	− 1.585	− 0.464	** < 0.001**
	2^a^	Intercept	1869	8.592 (7.119)	− 5.411	22.595	0.228
		Timepoint		0.060 (0.054)	− 0.047	0.167	0.268
		Sleep Quality		− 1.470 (0.339)	− 2.137	− 0.803	** < 0.001**
		Efficiency		0.119 (0.087)	− 0.053	0.291	0.174
		Duration		− 0.008 (0.008)	− 0.024	0.008	0.340
		Awakenings		0.074 (0.055)	− 0.035	0.183	0.182
	3	Intercept	1896	− 0.523 (6.982)	− 14.256	13.210	0.940
		Timepoint		0.053 (0.055)	− 0.055	0.162	0.332
		Efficiency		0.184 (0.088)	0.011	0.356	**0.037**
		Duration		− 0.012 (0.008)	− 0.028	0.005	0.162
		Awakenings		0.116 (0.055)	0.007	0.225	**0.037**
Pre-sleep fatigue	1	Intercept	1121	3.352 (0.244)	2.872	3.832	** < 0.001**
		Timepoint		0.043 (0.009)	0.025	0.061	** < 0.001**
		Sleep Quality		0.141 (0.064)	0.016	0.266	**0.027**
	2^a^	Intercept	787	3.778 (1.435)	0.954	6.601	**0.009**
		Timepoint		0.047 (0.010)	0.027	0.068	** < 0.001**
		Sleep Quality		0.093 (0.071)	− 0.046	0.232	0.187
		Efficiency		0.003 (0.017)	− 0.031	0.037	0.871
		Duration		0.000 (0.002)	− 0.003	0.003	0.853
		Awakenings		− 0.016 (0.011)	− 0.038	0.005	0.131
	3^a^	Intercept	787	4.360 (1.366)	1.673	7.047	**0.002**
		Timepoint		0.047 (0.010)	0.026	0.067	** < 0.001**
		Efficiency		− 0.001 (0.017)	− 0.034	0.032	0.953
		Duration		0.000 (0.002)	− 0.003	0.003	0.910
		Awakenings		− 0.018 (0.011)	− 0.039	0.003	0.092
Post-sleep fatigue	1	Intercept	983	1.673 (0.208)	1.264	2.083	** < 0.001**
		Timepoint		0.004 (0.008)	− 0.011	0.020	0.586
		Sleep Quality		0.647 (0.055)	0.539	0.755	** < 0.001**
	2^a^	Intercept	725	4.101 (1.320)	1.505	6.696	**0.002**
		Timepoint		0.012 (0.010)	− 0.007	0.031	0.218
		Sleep Quality		0.594 (0.065)	0.467	0.722	** < 0.001**
		Efficiency		− 0.028 (0.016)	− 0.059	0.003	0.079
		Duration		0.002 (0.001)	− 0.001	0.005	0.221
		Awakenings		− 0.033 (0.010)	− 0.053	− 0.013	0.001
	3	Intercept	803	7.771 (1.401)	5.015	10.527	< 0.001
		Timepoint		0.011 (0.011)	− 0.010	0.033	0.284
		Efficiency		− 0.053 (0.017)	− 0.087	− 0.019	0.002
		Duration		0.003 (0.002)	0.000	0.006	0.077
		Awakenings		− 0.045 (0.011)	− 0.067	− 0.023	< 0.001

Bold highlights *p < *0.05; *β *beta estimate; *p* significance value

*95% CI* *β* 95% Confidence Interval

^a^model of best fit

## Results

### Sleep over time

**Fig. 1:**
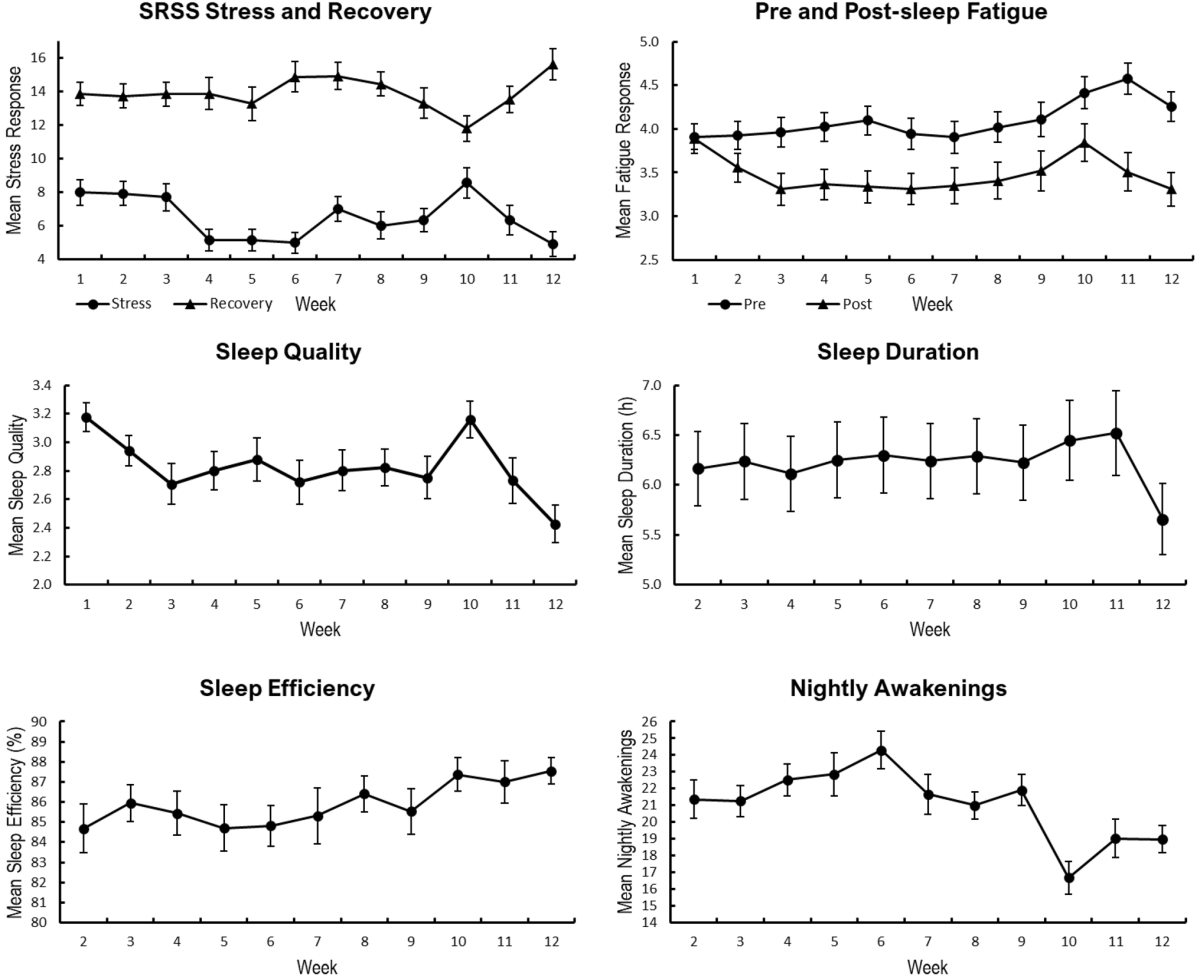
Mean (± SEM) subjective sleep quality, objective sleep metrics, SRSS stress and recovery and fatigue across the 12-week BMT program. *SRSS* Short recovery stress scale. A higher score indicates poorer sleep quality

Objective sleep metric daily means (± SD) over the 12-weeks were 6.3 h (± 1.2 h) sleep duration, 85.6% (± 5.5%) sleep efficiency, and 20.4 (± 7.6) awakenings (Fig. 1). A main effect of time was detected for mean weekly sleep efficiency, sleep duration, and awakenings as well as subjective sleep quality across 12 weeks BMT (*p < *0.05). Post-hoc analyses revealed subjective sleep quality was significantly poorer during week-1 than week-3, better during week-6 than week-10, poorer during week-10 than week-11, and better during week-12 than weeks 1, 2, 5, 7 and 10 (all *p < *0.05). Sleep duration was significantly lower during week-12 than all other weeks (*p < *0.05) except weeks 2 and 4. Sleep efficiency showed no significant differences between weeks. Number of Cole-Kripke awakenings was significantly higher during week-6 compared to weeks 8, 10 and 12; and lower during week-10 compared to weeks 4, 8 and 9 (all *p < *0.05).

### SRSS and fatigue over time

Main effects for time were present for SRSS stress and recovery, as well as pre- and post-sleep fatigue ratings (Fig. 1). Post-hoc analyses revealed SRSS stress responses were significantly higher at weeks 1, 2, 3, than weeks 4 and 6, higher at week-10 than weeks 4, 5 and 6, and higher at week-2 than week 12 (all *p < *0.05). SRSS recovery was significantly lower at week-10 than weeks 6, 7 and 12 (all *p < *0.05). Pre-sleep fatigue was significantly higher at week-11 than weeks 2 through 8 inclusive (*p < *0.05), while post-sleep fatigue was significantly higher at week-10 than week-12 (*p < *0.05).

### Relationships of sleep with stress, recovery, and fatigue

Subjective sleep quality showed the strongest relationships with stress, recovery and fatigue. It was most commonly a significant contributor to MLMs across all dependant variables and unlike objective metrics, was also a significant stand-alone predictor in single parameter models (all Model 1 of Table 2). Outside of pre-sleep fatigue, goodness of fit (AIC) was improved in every case by the inclusion of multiple independent variables in each iteration (Table 2). Details of the most effective multi-variable MLMs for sleep parameter relationships with stress, recovery and fatigue ratings are presented in Models 2 and 3 of Table 2, with Model 2 most commonly the best goodness of fit for parameters across all dependant variables.

For SRSS Stress, Model 2 had the best goodness of fit, where subjective sleep quality and objective sleep efficiency and awakenings showed significant relationships (all *p < *0.05; AIC = 1820). Marginally less effective was Model 3, with significant contributions of subjective sleep quality and objective sleep efficiency and awakenings (all *p < *0.05; AIC = 1826). For SRSS Recovery, Model 2 had the highest goodness of fit, although only including a significant individual relationship with subjective sleep quality (*p < *0.05; AIC = 1869). For pre-sleep fatigue, Models 2 and 3 showed equal AIC values: Model 2 (AIC = 787); Model 3 AIC = 787 (Table 2). Unlike other dependant variables, subjective sleep quality did not singularly have a statistically significant effect in pre-sleep fatigue models. Post-sleep Fatigue Model 2 had the best goodness of fit, including significant contributions of subjective sleep quality and objective awakenings (*p < *0.05; AIC = 725).

### Discussion

Weekly means of sleep efficiency, sleep duration, awakenings and subjective sleep quality changed significantly (*p < *0.05) over the 12-week BMT. Mean sleep duration across BMT was 6.3 (± 1.2 h) h. SRSS stress and recovery, as well as pre- and post-sleep fatigue ratings also changed significantly (*p < *0.05) over BMT. Relationships between sleep, and perceptions of stress, fatigue and recovery were best represented by sleep metrics of subjective sleep quality, objective sleep efficiency, objective sleep duration and objective awakenings. Subjective sleep quality showed the strongest goodness of fit relationships with stress, recovery, and fatigue.

### Sleep, stress, fatigue, and recovery during BMT

The present results demonstrate that there is significant week-to-week variation in objective sleep metrics, subjective sleep quality, stress, recovery, and fatigue during BMT. Of these measures, mean weekly objective sleep duration was the most stable of all measured variables throughout BMT (Fig. 1), however, it did not exceed 7 h per night. This is comparable to durations reported subjectively during U.S. BMT (Crowley et al. 2012; Ritland et al. 2021), and British BMT (Wentz et al. 2018). These sleep durations have the potential to cause a sleep debt in some recruits over a prolonged period, creating mood disturbance, and reducing occupational task performance and cognitive capacity (Scott et al. 2006). In support of our findings, previous research has shown that military training contexts similar to Australian Army BMT which include focused and sustained high workloads, could alter sleep, stress, fatigue and recovery metrics (Crowley et al. 2012).

Mean sleep efficiency across the 12-week training period was 85.6%, which is slightly higher than the threshold of 85% recognised by experts as indicative of good sleep quality (Ohayon et al., 2017). Nonetheless, as a mean value for the entire sample (*n* = 45), this still suggests many recruits had poor sleep quality (i.e., mean sleep efficiency < 85%) during the training period. Week-10 field training included nights of sleep deprivation, an outdoor sleeping environment and night activities (e.g., piquet), which appear to have negatively affected all measured sleep parameters, except for total sleep duration per 24 h compared to other weeks. While field training with night work negatively affected stress, fatigue and recovery, instructors were able to provide rest periods throughout entire 24 h periods to maintain approximately 6–7 h sleep. Sleep opportunity of 6–7 h during military field training appears to be atypical when compared to previous studies. For example, in a field exercise for British officer cadets, daily sleep time decreased from 5.6 ± 1.5 h a day in barracks, to 2.1 ± 1.3 h during field work (Needham-Beck et al. 2018); with an intentional reduction of sleep to 1 h opportunities during special forces training (Lieberman et al. 2006). Therefore, it appears the current Australian BMT field training period may be somewhat novel, recognising the importance of sleep and its impact on performance by allowing adequate sleep opportunities.

Of all self-reported measures in the current study, perceived stress exhibited the greatest week-to-week variability. This is consistent with previous research noting that elevations in stress during military training are common (Burchett et al. 2016), and in some cases desirable, when preparing recruits for the demanding nature of a military career. Occupational stress scores collected early in the training period in British junior entry recruits have effectively predicted attrition (Jackson et al. 2011), although notably, this was a younger cohort (age 16–17 years). However the current study is the first to quantify such measures weekly throughout the entire training period, where stress responses were elevated in weeks 1 through 3. These elevations during initial stages may partially explain observations that higher levels of perceived stress were associated with a greater risk of attrition in the current cohort of Australian Army recruits (Main et al. 2020). Notably, a reduction in stress responses was observed during week-4, when marksmanship training begins. Anecdotally this phase is considered significantly more ‘fun’ than the previous introduction (high pressure period), team building, marching and military skills foci of weeks 1 through 3. Week-10 field training caused the greatest increase in perceived stress of all weeks, which is similar to results from previous field training research (Lieberman et al. 2009).

Consistent with previous research, subjective post-sleep fatigue was lower than pre-sleep fatigue across BMT (Grandou et al. 2019), with the exception of week-1. The week-1 discrepancy is likely attributable, at least in part, to the initial response to the BMT environment (Crowley et al. 2012). Change in sleeping environment, and heightened stress and therefore stimulation upon arrival at BMT may have reduced restorative sleep and resulted in poorer post-sleep fatigue than later weeks. During field training of weeks 10 and 11, pre sleep fatigue was highest, which is consistent with previous studies reporting disrupted sleep resulting in fatigue during field training (Lieberman et al. 2006; Needham-Beck et al. 2018). Fatigue during week 12 was the lowest for BMT, suggesting that the training focus on ‘march-out’ preparations (i.e. the completion of BMT) provides a recovery opportunity for recruits following the field training phase.

Recruits perceived an increase in recovery at the ends of weeks 6 to 8, and 12. Weeks 6 and 12 are the programmed recovery weeks, with decreased physical demands. The week-6 recovery-period appears to allow recruits recovery from the weekly increase in physical training load, to a level where they cope with increased training load in the following weeks. This is congruent with non-linear training including periods of lower workload volume for recovery, which promotes adaptation; and is associated with an increased perception of recovery if successful (Abt et al. 2016). Whilst sleep opportunities were provided in week-10, awakenings due to the field sleeping environment and night time activities caused broken sleep cycles, and reduced perception of recovery, which has been previously reported (Vitale et al. 2019). This is a necessary part of preparing recruits for active duty. The perceived increase in recovery during week-12 suggests the intended recovery period prior to leaving BMT was effective.

### Relationships between sleep and stress, recovery, and fatigue

Goodness of fit for sleep metrics predicting stress, recovery and fatigue was improved in every case by the inclusion of multiple independent variables (Table 2). A MLM containing all of subjective sleep quality and objective efficiency, duration and awakenings (Model 2, Table 2) had the best goodness of fit with stress, recovery and fatigue. In the present analysis the most sensitive stand-alone measure to changes in stress, fatigue and recovery was subjective sleep quality. Further, subjective sleep quality was most commonly a significant contributor in the models of best fit, with the exception of pre-sleep fatigue, and had the greatest magnitude of effect on change of all dependant variables (Table 2; Sleep quality β Estimates). Sleep parameters are known to affect physical capacity and occupational performance in military populations (Grandou et al. 2019; Shattuck et al. 2018). Therefore, there could be utility in implementing subjective measures of sleep quality to monitor recruits’ adaptation to training loads, similar to what we see in elite sporting contexts. In these settings, sport scientists and researchers alike often prefer self-reported measures of training state for identifying those at risk of maladaptation and poor performance when undertaking high workloads (Saw et al. 2016).

While subjective sleep quality appears to be a very useful stand-alone predictor; augmenting with other objective sleep metrics improves the model’s goodness of fit between sleep metrics and SRSS Stress, SRSS Recovery, pre-sleep fatigue and post-sleep fatigue in BMT (Table 2 Model 1, sleep quality; vs. Model 2, sleep quality augmented with objective measures). The Model 2 MLM containing of sleep quality, efficiency, duration and awakenings (Model 2, Table 2) had the strongest associations with all dependant variables. Model 2 containing non-significant contributions from sleep variables still improved the goodness of fit, indicating most effective mediation of the dependant variable by a combination of co-variates. This is consistent with common psychophysiological monitoring strategies used in elite sports that recommend combining subjective and objective measures to ascertain the most holistic and accurate picture of an individual’s psychophysiological state, including sleep and recovery status (Halson 2014). The current results are the first to confirm that relationships of this nature are present in recruits during BMT.

The order of strength of association for each sleep metric with stress, recovery and post-sleep fatigue was subjective sleep quality, then objective sleep efficiency, awakenings then sleep duration (Table 2; β Estimates). Contrary to other dependant variables, pre-sleep fatigue did not include significant contributions from sleep quality and was equally well predicted by Model 2 and Model 3 (AIC = 787, Table 2). This indicates that mediation between the combination of variables is more important in this case than that of individual dependant variables. Further, pre-sleep fatigue is likely more affected by activities of the day, while post-sleep fatigue likely more affected by the night’s sleep. So a stronger link between sleep metrics and post sleep fatigue is expected.

Recent evidence indicates that insufficient sleep can become common for many military personnel over their career (Good et al. 2020), therefore developing poor sleep habits during BMT may also have downstream implications. While military work schedules a known to be demanding and can require periods of sustained wakefulness (Good et al. 2020), it is also widely recognised that both acute and chronic inadequate sleep durations and quality can lead to increased perception of stress as in this study, but also depressed mood states, and poor physical performance (Good et al. 2020; Tonon et al. 2020; Scott et al. 2006). These decrements could theoretically become multi-directional, as sleep causes poor performance, which in turn increases stress levels, then further adding to sleep difficulties. Poor work outcomes due to fatigue from poor sleep may further expose individuals to a medical or perceived need to utilise stimulants (Good et al. 2020), which could create dependencies and may further reduce individuals’ capacity for quality sleep outcomes if managed incorrectly. In this way, inadequate sleep is of great detriment to overall health, so making efficiently use of sleep opportunities is key for military personnel.

### Limitations, further research and practical implications

While compliance of correct activity monitor wear appeared high for all sleep periods, and data quality was very good, it is still possible that some participants may not have always worn their device correctly the entire night. The use of Likert scales for subjective assessment of sleep is common in the literature (Vincent et al. 2016); however, to our knowledge, such scales have not been validated against objective measures of sleep quality. As such, the extent to which subjective ratings of sleep quality in the current study reflected the actual sleep quality of participants is unclear. Nonetheless, a Likert scale was used because previously validated tools, such as the Pittsburgh Sleep Quality Index, ask users to reflect on their sleep during the previous month and thus are not designed for daily assessment of sleep quality (Buysse et al. 1989). The inherent potential bias within participants’ perception of their stress, fatigue and recovery is also recognised by the authors. However, all participants were aware that no Army personnel would see individual results, and anecdotally appeared to buy-in to the goal of improving the knowledge base for future recruits, and were comfortable to provide accurate assessments.

Further investigation is required to identify thresholds within subjective sleep quality or models such as Model 2 that indicate detrimental levels of stress, recovery or fatigue due to poor sleep. If thresholds indicating increased chance of poor outcomes for recruits are identified, options for recovery practice and protocols that are both feasible and effective in BMT could be tested, allowing eventual implementation of feasible, effective interventions for those who meet thresholds of poor sleep. This could potentially include a limited period of earlier bed-time to increase sleep opportunity. This should be effective as sleep efficiency is quite high, indicating the opportunity allowed is well utilised.

## Conclusion

Metrics of sleep, stress, fatigue and recovery all fluctuated across 12 weeks of BMT. The mean sleep duration of 6.3 h per night, may have a negative impact on training outcomes across BMT. A combination of subjective sleep quality and objective sleep efficiency, duration and awakenings appear to be most effective when explaining relationships between sleep and perceptions of stress, fatigue, and recovery during BMT. However, the most effective single predictor of perceived stress, recovery and fatigue was subjective sleep quality. If objective measures are not practical, subjective sleep quality is the best stand-alone option of variables tested in this study for tracking change in perception of stress, recovery or post-sleep fatigue during BMT.

## Data Availability

Data are not publically available but may be available from the corresponding author subject to further clarification and subsequent approval from the Australian Department of Defence.
